# Anatomic double-bundle medial patellofemoral ligament reconstruction with aperture fixation using an adjustable-length loop device: a 2-year follow-up study

**DOI:** 10.1186/s12891-018-2261-x

**Published:** 2018-09-25

**Authors:** Jae-Ang Sim, Jin-Kyu Lim, Byung Hoon Lee

**Affiliations:** 1 Department of Orthopaedic Surgery, Gil Hospital, Gachon University of Medicine and Science, Inchon, South Korea; 2 Department of Orthopedic Surgery, Kang-Dong Sacred Heart Hospital, Hallym University Medical School, 134-701, Gil-dong, Seoul, South Korea

**Keywords:** Medial patellofemoral ligament, Patella, Recurrent patellar dislocation, Double bundle, Aperture fixation, Adjustable-length loop device

## Abstract

**Background:**

To assess the clinical availability of an adjustable-length loop device for use in the double-bundle technique with aperture fixation at the patella and femur during anatomic double-bundle medial patellofemoral ligament reconstruction (DB-MPFLR) for recurrent patellar dislocation.

**Methods:**

We retrospectively investigated 11 patients (12 knees) with recurrent patellar dislocation who underwent anatomic DB-MPFLR with an ipsilateral semitendinosus tendon autograft. The graft was folded in half, and its central portion was hanged using the adjustable-length loop device. Both free ends of the graft were fixed at the proximal and distal ends of the medial edge of the patella by using suture anchors, and the hanged graft loop was pulled into the femoral tunnel while maintaining equal tension on both bundles. Manual traction of the suture loops was applied to fix the graft appropriately in full range of motion (ROM) of the knee joint under arthroscopic guidance. Clinical outcomes such as re-dislocation, ROM, clinical scores (Kujala score, Lysholm score, and visual analogue scale score for anterior knee pain), and complications were assessed preoperatively and at 2 years postoperatively. Radiographic parameters indicating patellar position, including congruence angle and lateral patellofemoral angle, were measured at 4 different angles of knee flexion (30°, 45°, 60°, and 90°).

**Results:**

At 4 different flexion angles of the knee joint, the preoperative congruence angle decreased significantly and the lateral patellofemoral angle increased significantly at the final follow-up (*P* <  0.001). Notably, the improvements in these angles were maintained with no significant differences at the 4 different flexion angles. None of the patients experienced subluxation or re-dislocation after surgery. The patellar instability symptoms improved, as confirmed on the basis of radiographic and other clinical outcomes.

**Conclusion:**

New DB technique with aperture fixation at the patella and femur by using an adjustable-length loop device offers high stability with full ROM of the knee joint, can be considered as a feasible procedure and technique for recurrent patellar dislocation.

## Background

Recurrent patellar dislocation (RPD) is related to various pathological abnormalities [1–10]. The medial patellofemoral ligament (MPFL) provides a primary restraint against the lateral dislocation of the patella [11, 12], and MPFL insufficiency is considered to be the main cause of traumatic RPD or patellar instability [13]. During MPFL reconstruction, graft fixation is critical to ensure the restoration of MPFL function. Several techniques have been introduced to fix the graft to the patellar MPFL attachment site, including the patellar bone tunnel technique [14–16] and the suture anchor technique [14, 17].

Non-anatomic reconstruction of the MPFL can lead to non-physiologic patellofemoral pressure and abnormal patellar tracking [18]. Therefore, recent techniques for reconstruction of the medial patellofemoral complex seek to restore the identical footprint of both the patellar and femoral attachments for biomechanical matching. The anatomic attachment site and anatomic shape of the native MPFL was previously defined. Double-bundle (DB) reconstruction at the patellar side may be a reasonable method for restoring the native ligamentous morphologic and biomechanical properties [17]. Therefore, increased interest has been directed toward anatomic DB reconstruction, which replicates 2 functional bundles, to more closely restore the normal patellofemoral stability and kinematics.

Nevertheless, the biomechanical rationale of anatomic DB reconstruction is not well established [19]. During DB anterior cruciate ligament reconstruction, both the anteromedial and posterolateral bundles are stretched and loaded in the extended knee position [20]. Therefore, graft fixation in this stretched and loaded position avoids elongation of the graft, potentially facilitating early rehabilitation with full range of motion (ROM). However, the DB MPFL reconstruction (DB-MPFLR) does not take into consideration the length change pattern of the respective bundles.

The aperture fixation technique introduced by Schӧttle et al. [21] may not apply the length change patterns at each knee flexion of the MPFL, a complex of functionally varying fibers, with some taut and others slack, throughout the range of knee motion [19]. Therefore, direct anatomic/aperture fixation [22] to restore the triangular form of the MPFL for anatomic reconstruction can result in uneven and non-isometric graft tensioning and might induce non-physiologic patellofemoral loads and kinematics [23] with full ROM. Furthermore, micro-motion of the graft during knee flexion-extension can increase the risk of delayed or insufficient tendon-to-bone healing [24].

We hypothesized that the adjustable-length loop device used in femoral cortical suspension systems, which are the most convenient devices for use in ligament reconstruction with soft tissue graft [25], will be applicable in anatomic DB-MPFLR, with 2 clinical benefits. First, certain reciprocal movement of the looped graft into the femoral tunnel may allow the even tension to restrain the lateral force throughout the ROM. Second, graft fixation with appropriate tension in full ROM of the knee joint can be easily achieved by manual traction using lead sutures under arthroscopic guidance. Here, we describe a DB technique with aperture fixation at the patella and femur by using an adjustable-length loop device, which offers high stability in full ROM of the knee joint.

## Materials and methods

Between 2014 and 2015, 18 patients underwent surgery for the treatment of RPD depending on individual pathologic abnormalities. All surgeries were done by the same senior orthopedic surgeon. All patients who underwent surgery during this period were screened. The indication for operation was RPD (defined as at least 2 episodes of patellar dislocation despite non-operative treatment). Lateral patellar dislocations were diagnosed on the basis of history taking, physical examination, simple radiographs, computed tomography, and magnetic resonance imaging. Seven patients who required additional procedures for RPD and had various pathologic abnormalities [1, 2], such as bony pathologies on the femoral or tibial side, were excluded, as follows: trochlear dysplasia (Dejour classification C) [3, 4, 26] (*n* = 1), increased tibial tuberosity to trochlear groove (TT-TG) distance (> 20 mm) [4, 5] (*n* = 4), patella alta (Insall-Salvati [IS] ratio > 1.5) [27] (*n* = 1), and combined genu valgum deformity (criteria: > ± 3° mechanical femorotibial angle [MFTA] on anteroposterior long-leg weight-bearing lower-extremity scanographs) [28] (*n* = 1). Patellar height, TT-TG distance, and MFTA were preoperatively assessed by the same surgeon. Patients with a minimum postoperative follow-up of 2 years were considered eligible. Eventually, a total of 11 patients (12 knees) with RPD were treated using our approach of anatomic DB-MPFLR. The current study obtained institutional review board approval (GAIRB 2017–236) before the study onset, and informed consent was obtained from all patients. The patients’ demographic data are presented in Table 1.

**Table 1:** Demographic Data on Patients

No	Sex/Age	Episode	Patellar position	F/u period (months)	MFTA (^o^)	TT-TG distance (mm)	IS Ratio^a^		ROM (^o^) ^a^		pVAS score^a^		Kujala score^a^		Lysholm score^a^	
							Preop	Postop	Preop	Postop	Preop	Postop	Preop	Postop	Preop	Postop
1	F/18	2	SL	25	1.6	17.1	1.2	1.1	130	130	6	3	54	88	70	91
2	F/13	4	SL	26	0.6	11.2	1.4	1.0	135	140	4	0	62	94	65	99
3	M/14	3	SL	26	2.7	12.2	1.1	1.1	130	130	5	1	75	98	70	100
4	M/16	2	SL	25	−3.0	10.2	1.3	1.2	130	130	5	2	56	90	75	90
5	F/28	> 10	SL	24	1.1	16	1.0	0.9	120	125	7	2	56	84	74	85
6	F/16 (Lt)	2	SL	24	0.7	10.1	1.1	1.1	150	150	4	1	70	86	72	90
	(Rt)	2	SL	26	−1.7	15.4	1.2	1.0	140	140	4	1	72	86	70	90
7	M/22	2	SL	35	1.1	14.5	1.2	1.1	130	130	6	2	75	92	72	94
8	M/19	5	SL	34	3.0	19.9	1.0	1.0	130	130	4	3	80	98	76	100
9	F/26	4	SL	31	−2.7	13.1	1.4	1.3	135	130	5	0	63	93	76	100
10	M/18	2	SL	36	−3.0	18.2	1.1	1.0	120	125	3	1	75	95	70	95
11	F/18	2	SL	27	−0.2	16.2	1.3	1.3	135	135	3	0	70	80	70	85

*IS Ratio* Insall-Salvati ratio, *MFTA* mechanical femorotibial angle, *pVAS score* Visual analogue scale for the anterior knee pain, *ROM* range of motion, *SL* Subluxation, *TT-TG* tibial tuberosity to trochlear groove

^a^IS Ratio and clinical scores (Kujala, Lysholm, and pVAS score) were evaluated at preoperatively and postoperative 2-year follow-up

### Surgical technique

**Fig. 1:**
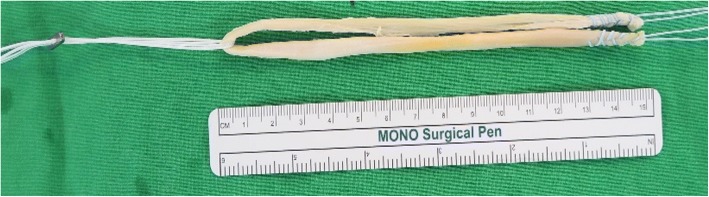
Graft preparation

Before RPD correction, diagnostic arthroscopic examination was performed in all patients. After the completion of arthroscopy, a 2-cm-long oblique incision was performed at the pes anserinus. After incising the sartorius aponeurosis, the semitendinous tendon was harvested and used as an autograft. The usable part of the tendon needed to be at least 20 cm long. After the tendon was harvested using a stripper and the muscle tissue was removed, the doubled tendon diameter was determined and both ends were whip stitched using an absorbable braided suture over a length of 15 mm. The graft was then folded in half, and its central portion was hanged using the adjustable-length loop device (TightRope RT; Arthrex Inc., Naples, FL, USA). The diameter of all doubled tendons was 7 or 8 mm (Fig. 1).

### Preparation for patellar fixation

**Fig. 2:**
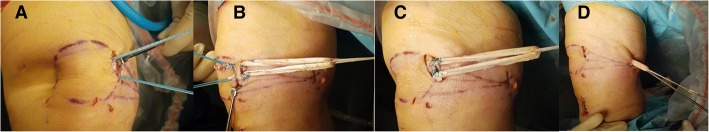
A 2-cm longitudinal incision was made at the medial border of the patella. The deep fascia and periosteum were detached and reflected medially. After minimal decortication of the reconstruction area, 2 suture anchors were inserted into the proximal margin and center of the medial aspect of the patella (**a**). The free graft ends were sutured to the inserted grafts after flipping them over through the detached periosteum (**b** and **c**). Looped graft with the adjustable-length loop device at the ending was brought into the separated layer between the vastus medialis obliquus (VMO) and the joint capsule, using long curved Kelly with caution to avoid any injury to the joint (**d**)

A 2-cm incision was made at the medial border of the patella. The superomedial aspect of the patella was approached. To achieve aperture fixation on the patellar side, the free graft ends were directly fixed to the patella. A longitudinal periosteal incision was made about 1 cm lateral from the medial borderline of the patella, and the periosteum was detached and reflected medially. After the MPFL footprint was exposed, minimal decortication of the reconstruction area was performed for better bone-to-graft healing. Two guidewires were drilled tangentially into the patella at the proximal and distal ends of the medial edge, and 2 suture anchors (Bio Mini-Revo®; Linvatec, Largo, FL, USA) were inserted into the proximal margin and center of the medial aspect of the patella (Fig. 2). The free graft ends were sutured to the inserted grafts after flipping them over through the detached periosteum. Thereafter, the medial patellar periosteal tissue was sutured, covering the embedded graft and avoiding subcutaneous irritation by the knots.

### Femoral tunneling and graft tensioning

**Fig. 3:**
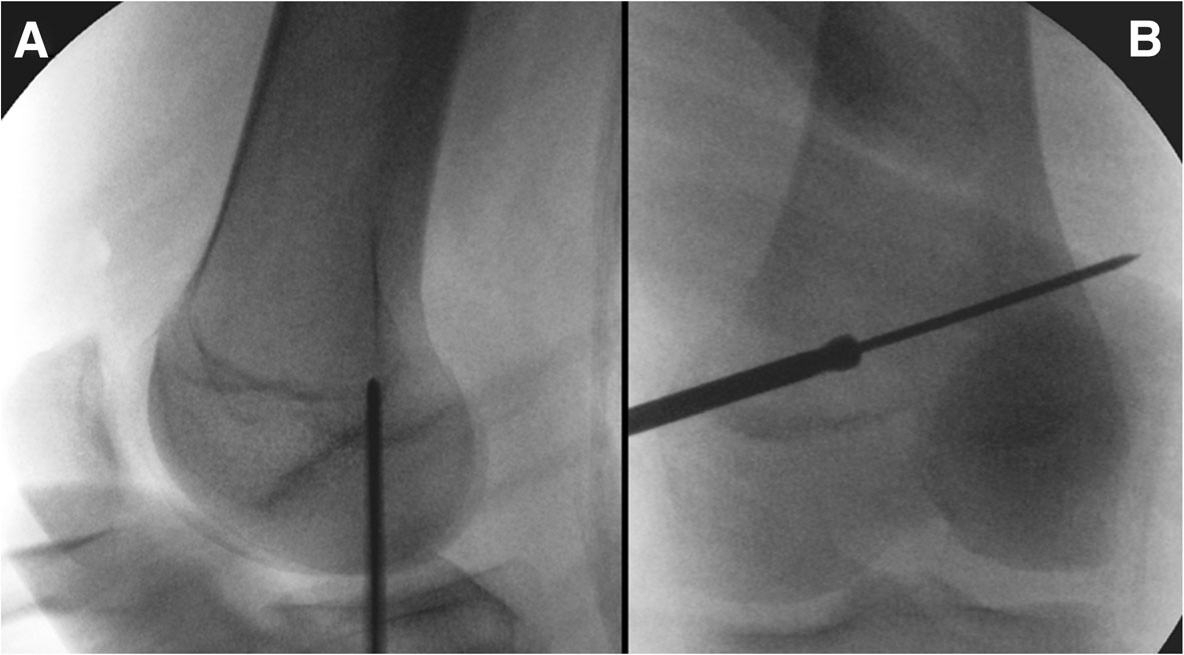
A femoral tunnel was made at the Schöttle point [2] (**a**) in the proximal and anterior direction in order to prevent iatrogenic peroneal nerve injury. **b** Intraoperative control was achieved using an image intensifier

**Fig. 4:**
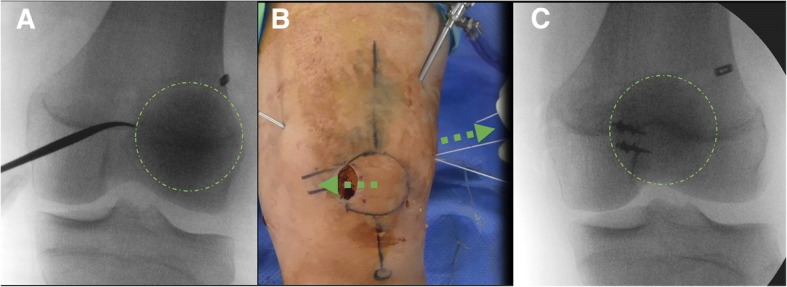
The patellar position was tracked and fixed simultaneously by manual traction of the suture loops (**b**), and the graft was appropriately fixed in full range of motion of the knee joint with the lateral patellar edge positioned in line with the lateral trochlear border, under arthroscopic guidance (**a**, **c**)

In each femur, the femoral tunnel was made at the Schöttle point [29] in the proximal and anterior direction under C-arm guidance, in order to prevent iatrogenic peroneal nerve injury (Fig. 3a). Then, the tunnel was created to pass the button of the adjustable-length loop device, for which a 4.0-mm cannulated reamer was used. The length of the tunnel was measured using a depth gauge, and the femoral tunnel was drilled to have the same diameter as the graft until there was an 8-mm bone stock from the lateral femoral cortex (Fig. 3b). The graft was pulled into the femoral tunnel by using the lead suture inserted from the outside to the inside of the tunnel while maintaining equal tension on both bundles. The 2 strands of the graft passed between the first and second layers. The graft was fixed using the button of the device after it was flipped over the lateral cortex. After confirming that the button was on the cortex, manual traction of the suture loops was applied to fix the graft appropriately in full ROM of the knee joint with the lateral patellar edge positioned in line with the lateral trochlear border, under arthroscopic guidance (Fig. 4). The lateral retinaculum was released if the patient experienced lateral tightness. By using electrocautery under arthroscopic visualization, the capsular structure was released longitudinally along the lateral margin of the patella. The release was performed from 2 cm proximal to the superior patellar pole and extended distally for 1.5 to 2 cm.

### Postoperative rehabilitation

Tolerable weight bearing was allowed and quadriceps setting exercises could be started immediately with free ROM, if tolerated. Running or cycling was permitted at 6 weeks after the operation; full activity was permitted at 3 months after the operation.

### Outcome evaluation

Clinical outcomes related to recurrence of dislocation, apprehension test, ROM, clinical scores (Kujala score [30], Lysholm score [31], and visual analogue scale for anterior knee pain [pVAS]), complications, and radiological outcomes (congruence angle, lateral patellofemoral angle [32], and IS ratio) were assessed before and after the operation, and at the final 2-year follow-up.

Our primary outcomes of interest were radiographic parameters indicating patellar position, including congruence angle [2] and lateral patellofemoral angle [32]. The sulcus angle is the angle between a line between the lateral femoral condyle and intercondylar sulcus midpoint and a line between the medial femoral condyle and intercondylar sulcus midpoint. Each parameter was measured at 4 different angles of knee flexion (30°, 45°, 60°, and 90°).

#### Statistical considerations

Statistical tests were performed using IBM SPSS version 22 (IBM, Armonk, NY, USA). Continuous variables were described as mean ± standard deviation (SD). A priori power analysis was performed to determine the sample size with the 2-sided hypothesis test considering an α error of 0.05 and power of 0.90. The calculations involving our sample size of 11 patients indicated adequate power (0.85–0.95) to detect a significant difference of 5 degrees in the measurement outcomes of congruence angle and lateral patellofemoral angle in the present study. Wilcoxon signed-rank tests were used to compare the pVAS, Lysholm, and Kujala scores; IS ratio; congruence angle; and lateral patellofemoral and sulcus angles before and after the operation. A *P*-value of < 0.05 was considered statistically significant. The intra-class correlation coefficient (ICC) was determined to rule out observation bias between the 2 separate orthopedic surgeons. The parameters were measured twice, at an interval of 2 weeks.

## Results

The patients’ mean age at surgery was 18.6 ± 4.4 years (range: 13–28 years). The median follow-up period was 28.8 months (range: 24–48 months). The average value of the IS ratio was 1.2 (SD: 0.1) and the TT-TG distance was 14.5 cm (SD: 3.2). All measured ICCs were almost good to perfect, ranging from 0.726 to 0.991.

At the time of the final assessment, ROM was restored to the preoperative level and anterior knee pain improved in all patients, indicated by a decrease in the mean pVAS score from 4.7 ± 1.2 to 1.3 ± 1.1 (*P* <  0.001). No patients experienced surgical complications, including patellar fracture and re-dislocation. The patellar instability symptoms improved, as confirmed on the basis of the radiographic outcomes as well as the Lysholm and Kujala scores. The mean Lysholm score improved from 71.7 ± 3.2 preoperatively to 93.3 ± 5.6 postoperatively (*P* <  0.001), and the Kujala score improved from 67.3 ± 8.8 preoperatively to 90.3 ± 5.7 postoperatively (*P* <  0.001) (Table 1).

**Fig. 5:**
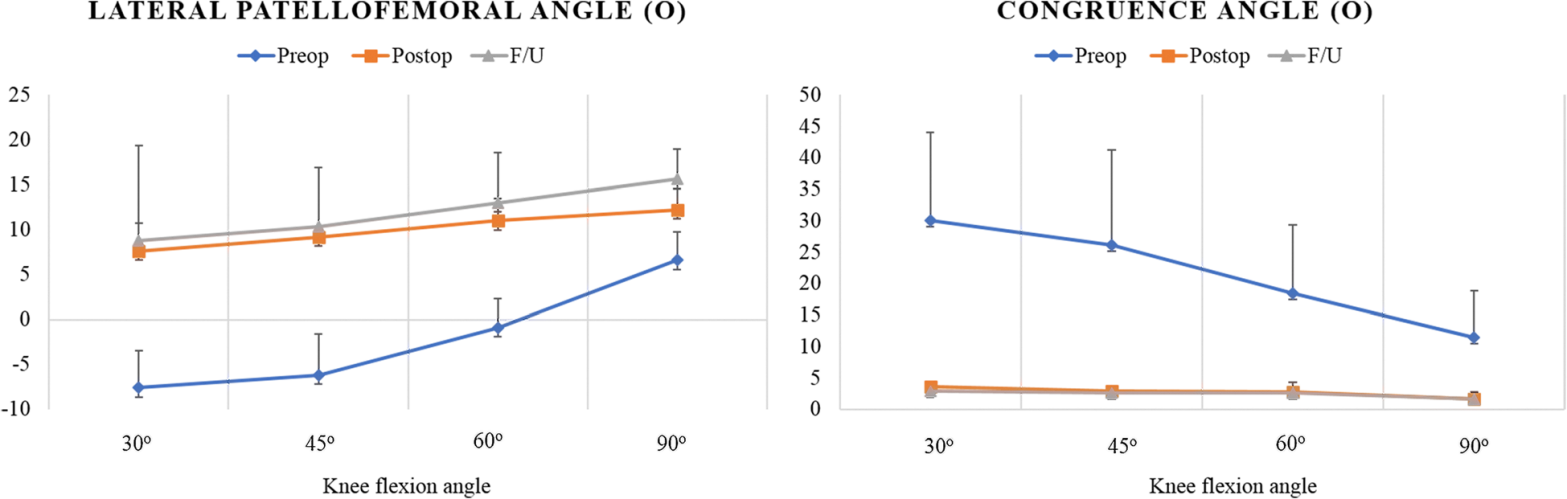
Changes in the congruence and lateral patellofemoral angles after the surgery

On merchant view with the patient supine, the knees flexed 30 degrees [33], the preoperative lateral patellofemoral angle (− 7.6 ± 10.6 to 7.6 ± 3.1, *p* <  0.001) and congruence angle (30.1 ± 13.9 to 3.6 ± 1.5, *p* <  0.001) were improved after reconstruction. The congruence angle and the lateral patellofemoral angle were also improved significantly at 4 different flexion angles (30^o^, 45^o^, 60^o^, and 90^o^) of the knee joint (*p* < 0.001). The improvements in these angles were maintained with no significant differences at the 4 different flexion angles (Table 2) (Fig. 5). Analysis of the measured radiographic parameters showed that patellar height, determined from the IS ratio, decreased slightly from 1.2 ± 0.1 to 1.1 ± 0.1 after the operation (*P* < 0.001).

**Table 2:** Preoperative, Immediate Postoperative, and Two-year Follow-Up Radiologic Parameters^a^

Knee flexion angle		Preop		Postop		F/U
Lateral patellofemoral angle (^o^)	30 ^o^	−7.6 ± 10.6		7.6 ± 3.1		8.8 ± 4.1
			***< 0.001***		*0.260*	
	45 ^o^	−6.2 ± 6.5		9.2 ± 0.4		10.4 ± 4.6
			***0.001***		*0.285*	
	60 ^o^	−0.9 ± 5.6		11.0 ± 2.5		13.0 ± 3.2
			***0.006***		*0.140*	
	90 ^o^	6.6 ± 3.4		12.2 ± 2.4		15.6 ± 3.2
			***0.033***		*0.093*	
Congruence angle (^o^)	30 ^o^	30.1 ± 13.9		3.6 ± 1.5		2.9 ± 1.3
			***< 0.001***		*0.221*	
	45 ^o^	26.1 ± 15.2		2.9 ± 1.0		2.7 ± 1.0
			***0.001***		*0.233*	
	60 ^o^	18.4 ± 11.0		2.8 ± 1.6		2.7 ± 1.4
			***0.014***		*0.008*	
	90 ^o^	11.5 ± 7.4		1.7 ± 1.1		1.7 ± 1.0
			***0.010***		*0.483*	
Sulcus angle (^o^)	30 ^o^	146.9 ± 7.4		145.3 ± 6.4		145.4 ± 5.5
			*0.491*		*0.442*	
	45 ^o^	146.6 ± 2.4		145.5 ± 1.7		146.0 ± 4.8
			*0.549*		*0.255*	
	60 ^o^	146.1 ± 4.5		145.2 ± 5.9		146.5 ± 5.4
			*0.870*		*0.302*	
	90 ^o^	146.4 ± 3.4		145.8 ± 5.5		144.8 ± 5.0
			*0.634*		*0.322*	
IS Ratio		1.2 ± 0.1				1.1 ± 0.1
				***< 0.001***		

*IS Ratio* Insall-Salvati ratio

^a^Value are mean ± standard deviation

*P*-value was expressed in Italic

Values of *P* < 0.05 are displayed in bold

## Discussion

The most important findings of the present study were that the aperture fixation technique using the adjustable-length loop device from femoral cortical suspension systems improved the alignment parameters (congruence angle and patellar tilt angle) and yielded high stability in full ROM of the knee joint for 2 years after the operation.

Reconstruction techniques with the DB structure for restoring the anatomic shape of the MPFL have been recently highlighted [34, 35]. The MPFL is not a single-bundle structure but a complex of functionally varying fibers, some of which are taut and some are slack, throughout the range of knee motion [19]. Superior patellar fixation may cause patellar instability at mid to high knee angles, and conversely, inferior patellar fixation may produce excessive laxity at low flexion angles [19]. Anatomic DB-MPFLR lowers patellar rotation during the flexion-extension movement that may occur during single-bundle reconstruction.

Undoubtedly, the aperture fixation technique has clinical benefits in terms of the effort required to decrease the risk of delayed or insufficient tendon-to-bone healing [21, 24]. Schӧttle et al. indicated that it provides high stability, as the proximal bundle seems to stabilize in extension while the distal bundle stabilizes in flexion.

However, as described in the Introduction, we hypothesized that certain reciprocal movement of the looped graft into the femoral tunnel may ensure more biocompatible reconstruction, rather than leaving the tension of each bundle to the discretion of the surgeon without a thorough understanding of the length change or the biomechanics of the 2 bundles of the MPFL throughout the range of knee motion. We did not directly evaluate the permissible amount of the reciprocal movement of the looped graft into the femoral tunnel. We sought to determine the clinical significance of the technique on the basis of our finding that improvements in radiographic parameters representing patellar position were noted irrespective of the angle of knee flexion (30°, 45°, 60°, or 90°). We also found that at the 2-year follow-up, no graft slackening, graft failure, or reduction failure from elongation of the graft or the “bungee” effect in the femoral socket had occurred in any case. However, it remains to be determined whether a kinematic isometric length change of about 6–9 mm throughout the range of knee motion [19] affects the radiological and clinical outcomes in clinical practice.

Other studies described several techniques for anatomic DB-MPFLR. White and Sherman [17] used an absorbable soft tissue interference screw for femoral fixation in 30° of knee flexion. Colvin and West [36] also used absorbable interference screws for femoral fixation of the 2 free ends of the graft in 30° of knee flexion, and performed combined bone groove and suture anchor fixation at the patella. Dejour et al. [37] used a “Y”-shaped graft for reconstruction. An absorbable soft tissue interference screw was used for femoral fixation, and the lateral bone bridge tie was used for patellar fixation. Additionally, they introduced the surgical technique of lateral retinaculum plasty to release eccentric dynamic loading through the ROM.

Graft fixation is recommended with appropriate tension at 20–30° of knee flexion and with the patella aligned in the trochlear groove throughout the entire ROM of the knee. However, it is not easy to determine the appropriate graft tension during graft fixation. Previous arbitrary techniques that rely on the surgeon’s subjective skill carry the risk of over-tensioning of the MPFL graft, which can increase the patellofemoral pressure [38]. Goutallier et al. [39] reported that anterior knee pain persisted after the operation in up to 38–40% of patients. Our technique using the adjustable-length loop device has clinical applicability in this aspect. The patellar position, which can easily be determined under arthroscopic guidance throughout flexion, was tracked and fixed simultaneously by a simple pulling of the suture loops. Moreover, the possible reciprocal movement of the looped graft into the femoral tunnel might release eccentric loading through the ROM. It can also diminish the learning curve to achieve appropriate graft tensioning during MPFL reconstruction. In the present study, we found that anterior knee pain was relieved in all patients, the patients had full ROM immediately after the surgery, and there was no reduction loss at follow-up with early rehabilitation.

Our study has some inherent limitations because of its retrospective design. The relatively short follow-up period and small sample size were also limitations in judging the postoperative outcomes. However, a 2-year follow-up period is sufficient to determine clinical outcomes such as re-dislocation. Furthermore, a lack of comparison with other aperture techniques is a major limitation of the present study. In addition, this study did not directly compare the patellofemoral kinematic changes by using additional biomechanical measurement tools. Thus, the findings may not be adequate to determine the clinical relevance of using the adjustable-length loop device with regard to patellofemoral kinematics.

## Conclusion

New DB technique with aperture fixation at the patella and femur by using an adjustable-length loop device offers high stability with full ROM of the knee joint, can be considered as a feasible procedure and technique for recurrent patellar dislocation.

## Abbreviations

ACLR: Anterior cruciate ligament reconstruction
AP: Anteroposterior
DB-MPFLR: Double-bundle medial patellofemoral ligament reconstruction
ICC: Intra-class correlation coefficient
MFTA: Mechanical femorotibial angle
MPFL: Medial patellofemoral ligament
pVAS: Pain visual analogue scale
ROM: Range of motion
RPD: Recurrent patellar dislocation
SD: Standard deviation
TT-TG: Tibial tuberosity to trochlear groove
